# Early Maladaptive Schemas as Core Therapeutic Targets in Eating Disorders and Obesity: A Schema Therapy–Informed Network Analysis

**DOI:** 10.1002/cpp.70153

**Published:** 2025-09-22

**Authors:** Matteo Aloi, Marianna Rania, Elvira Anna Carbone, Renato de Filippis, Ettore D'Onofrio, Lavinia Rotella, Daria Quirino, Susan Simpson, Cristina Segura‐Garcia

**Affiliations:** ^1^ Department of Clinical and Experimental Medicine University of Messina Messina Italy; ^2^ Outpatient Unit for Clinical Research and Treatment of Eating Disorders University Hospital Renato Dulbecco Catanzaro Italy; ^3^ Department of Health Sciences University “Magna Graecia” of Catanzaro Catanzaro Italy; ^4^ Department of Medical and Surgical Sciences University “Magna Graecia” of Catanzaro Catanzaro Italy; ^5^ UniSA Justice & Society University of South Australia Adelaide South Australia Australia; ^6^ NHSForth Valley Larbert UK

**Keywords:** network analysis, person‐centred approach, psychotherapy, Schema therapy, treatment

## Abstract

Early maladaptive schemas (EMSs) are stable cognitive‐emotional patterns central to the psychopathology of eating disorders (EDs) and obesity. This study used a schema therapy–informed network analysis to explore the structure and significance of EMSs in people with anorexia nervosa (AN, *n* = 129), bulimia nervosa (BN, *n* = 124), binge‐eating disorder (BED, *n* = 166), and obesity (*n* = 152). Networks were modelled using the Young Schema Questionnaire (YSQ‐S3), with expected influence as the main centrality, in a final sample of 571 patients. In AN, the most central schemas were Defectiveness/Shame, Negativity/Pessimism, and Subjugation, reflecting self‐criticism, hopelessness, and submission. BN showed a similar pattern with high centrality for Subjugation, Defectiveness/Shame, and Negativity/Pessimism. In BED, central EMSs included Subjugation, Social Isolation, Negativity/Pessimism, and Abandonment, emphasizing loneliness and emotional vulnerability. The obesity group showed dominant roles for Negativity/Pessimism, Social Isolation, Subjugation, and Mistrust/Abuse, indicating patterns of mistrust, hopelessness, and relational avoidance. Negativity/Pessimism and Subjugation were central across all groups, highlighting their transdiagnostic relevance. Interestingly, Social Isolation appeared prominently in BED and obesity, possibly associated with earlier experiences of bullying and rejection. These findings underscore the clinical value of targeting central EMSs in schema therapy, offering a network‐based framework to guide individualized interventions across EDs and obesity.

## Summary

- Schema therapy offers a valuable framework for understanding and targeting the cognitive‐emotional underpinnings of eating disorders (EDs) and obesity by focusing on early maladaptive schemas (EMSs).
- Subjugation and Negativity/Pessimism emerged as core transdiagnostic schemas across all ED and obesity groups, highlighting the importance of addressing themes of emotional surrender and hopelessness in treatment.
- Disorder‐specific schemas such as Defectiveness/Shame in anorexia and bulimia, and Social Isolation and Abandonment in binge‐eating disorder and obesity, suggest the need for tailored schema‐based interventions.
- Network analysis provides a person‐centred, empirically grounded approach to identify and prioritize therapeutic targets in complex clinical profiles, enhancing the precision and effectiveness of schema‐focused treatments.

## 1 Introduction

Schema therapy (ST) is an integrated, empirically supported psychotherapy that combines elements of cognitive‐behavioural, attachment, psychodynamic, and experiential models to address underlying psychological patterns rooted in childhood experience (Young et al. 2003). Central to ST is the theory of Early Maladaptive Schemas (EMSs), defined as enduring and self‐defeating themes or patterns that develop during childhood or adolescence, most often in response to unmet emotional needs (Young et al. 2003). These schemas are constructed across the lifespan and transform into maladaptive lenses through which individuals see themselves, others, and the world (Young et al. 2003).

ST has been found to have positive results in the treatment of EDs with mounting evidence supporting its use in varying diagnostic presentations. Literature accounts for EDs as stemming from childhood trauma and interpersonal relationships, with schema modes and the ‘eating disorder voice’ being identified as playing a central role in maintaining disordered eating patterns (Cunningham et al. 2024). Schema therapy of EDs entails the identification and recovery of these schemas (Simpson and Smith 2019), operating on issues behind food and body cognitions of the sort that are the target of cognitive‐behavioural therapy (Fairburn 2008). ST has also been discovered to enhance awareness, support adaptive modes, and decrease maladaptive modes, leading to improved ED patient outcomes (Cunningham et al. 2024). By addressing core beliefs and childhood experiences, ST offers a consolidated model of treatment and understanding for EDs that may benefit individuals who have not responded to standard approaches (Joshua et al. 2023).

Empirical support from the recent systematic review by Maher et al. (2022) reiterates the pivotal position of EMSs in the description of EDs. ED patients across diagnoses consistently report considerably higher EMS scores than both nonclinical and clinical controls. While most EMSs are elevated in all EDs, some schemas become particularly prevalent within specific diagnostic groups, with implications for disorder‐specific schema profiles.

In fact, EMS trajectories are different between ED subtypes and associated behaviours. Patients with Bulimia nervosa (BN) or Binge‐eating disorder (BED) are likely to score more in schemas of insufficient self‐control, emotional deprivation, grandiosity or entitlement, impulsivity, abandonment, and harm vulnerability (Basile et al. 2019; Rania et al. 2020), whereas persons with restrictive EDs, such as anorexia nervosa (AN), are likely to score on social undesirability, failure, subjugation, unrelating standards, self‐sacrifice, and punitiveness schemas (Pauwels et al. 2016; Pugh 2015). These varied schema profiles highlight the significance of treatment for pathological rigidity of the underlying beliefs that fuel ED symptoms and behaviours to encourage remission.

But little is understood concerning how they interact with each other and which ones are most instrumental in the perpetuation of EDs and psychological problems related to obesity. Classic statistical methods tend to isolate schemas, neglecting their possible interconnections. Network analysis provides a valuable alternative in that it simulates the dynamic interactions among EMSs as a system, facilitating an examination of the most impactful schemas within and between diagnostic categories (Borsboom 2022; Borsboom and Cramer 2013; McNally 2016).

The purpose of this study is to examine the structure of the network and centrality of EMSs in AN, BN, BED, and OB. Under a schema therapy–guided network approach, we fit individual models for each condition based on the EMSs according to Young Schema Questionnaire—Short Form 3 (YSQ‐S3). Through the identification of both group‐specific and transdiagnostic schemas, this research seeks to inform more accurate and beneficial schema‐based interventions for the EDs‐obesity spectrum.

## 2 Methods

### 2.1 Participants

Participants eligible for inclusion were selected among those seeking care in the Outpatient Unit for Clinical Research and Treatment of Eating Disorders at the University Hospital ‘Renato Dulbecco’ of Catanzaro (Italy) between June 2018 and June 2025. They were consecutively recruited during their initial visit for participation in this cross‐sectional study, and the aim and the description of the research were presented by the research team. To be included, patients needed to be aged between 14 and 65, diagnosed with an ED according to DSM‐5 criteria (American Association Psychiatric 2013), willing to take part, and able to provide valid informed consent. In addition, for the obesity group, patients were included if they had a body mass index (BMI) ≥ 30, thus covering the full spectrum of obesity severity (Class I to Class III). This allowed us to capture the entire continuum of eating‐related presentations, ranging from restrictive profiles to obesity. Exclusion criteria included comorbidity with severe psychiatric diagnoses (e.g., neurodevelopmental, schizophrenia spectrum, bipolar disorders, and neurocognitive disorders), neurological or medical conditions (e.g., diabetes), active substance dependence or abuse (within ≤ 6 months), and other medical comorbidities or treatments that could influence eating behaviours.

Each participant underwent a diagnostic interview conducted by experienced psychiatrists through the Structured Clinical Interview for the DSM‐5 (SCID‐5‐CV) (First 2016) and the Eating Disorder Examination (EDE 17.0D) (Calugi et al. 2015).

Out of the 602 patients initially approached for the study, 31 (5.0%) were screened out on the following reasons: nine patients (1.5%) were not eligible due to current substance use disorder; nine (1.5%) did not finish the assessment and hence were excluded; seven (1.2%) had intellectual disability exclusion criteria; and seven (1.2%) were excluded because they had psychotic symptoms. Accordingly, the ultimate sample consisted of 571 patients (*N* = 129 with anorexia nervosa–restricting subtype [AN‐R], *N* = 124 with bulimia nervosa [BN], *N* = 166 with binge‐eating disorder [BED], and *N* = 152 with obesity without an eating disorder diagnosis [OB]).

Only the patients who accepted to participate in the research protocol provided informed consent, and completed the evaluation were included in the analysis. No missing data were reported in the participants' socio‐demographic information or in the assessment. This study adhered to the ethical principles outlined in the updated Helsinki Declaration (World Medical Association 2013) and received approval from the Ethical Committee of ‘Regione Calabria, Sezione Area Centro’ (identifier: Prot. 66/15.03.2018). Before completing the questionnaires, participants provided written informed consent. For minors, consent was acquired from their parents or legal tutors after providing detailed information.

### 2.2 Measures

- Eating Disorder Examination (EDE): This clinical interview measures the extent and severity of eating psychopathology on four subscales with 28 questions: Eating Restraint, Eating Concern, Weight Concern, and Shape Concern, determining a global EDE score (Calugi et al. 2015). The semi‐structured interview assesses ED‐related behaviour and psychopathology in the last 3 months. It explores behavioural symptoms of EDs, including binge eating, induced vomiting, diuretic and laxative abuse, excessive exercise, and restriction of food. Higher scores reflect an increased level of severity of psychopathology. Internal consistency of the EDE‐Q subscales and global score was assessed using McDonald's omega (*ω*), which is considered a robust alternative to Cronbach's alpha. The omega values indicated good to excellent internal reliability across the subscales. Specifically, the Eating Restraint subscale showed an *ω* of 0.84, Eating Concern *ω* = 0.78, Shape Concern *ω* = 0.92, and Weight Concern *ω* = 0.88. The total score demonstrated excellent internal consistency, with an omega of 0.94.
- Young Schema Questionnaire Short Form‐3 (YSQ‐S3). The YSQ‐S3 contains 90 items measured on a 1 (*not at all false for me*) to 6 (*describes me exactly*) Likert scale to evaluate the presence of the 18 early maladaptive schemas (EMSs) according to Young and colleagues' theory (Young and Brown 2018). Higher scores indicate higher levels of EMSs. Under a newly introduced model by Bach and others (Aloi, Rania, Sacco, et al. 2020; Bach et al. 2018), YSQ‐S3's 90 items are contained in 18 EMSs under four domains: (1) Disconnection and Rejection; (2) Impaired Autonomy and Performance; (3) Excessive Responsibility and Standards; (4) Impaired Limits. The internal consistency of the 18 early EMSs of the YSQ‐S3 was examined using McDonald's *ω*. Overall, reliability coefficients were acceptable to excellent across most schemas, ranging from 0.699 to 0.893. The highest reliability values were observed for Failure (*ω* = 0.888), Defectiveness/Shame (*ω* = 0.874), and Social Isolation (*ω* = 0.859). Good reliability was also found for Mistrust/Abuse (*ω* = 0.850), Emotional Deprivation (*ω* = 0.844), Negativity/Pessimism (*ω* = 0.840), Abandonment (*ω* = 0.831), Self‐Sacrifice (*ω* = 0.824), and Emotional Inhibition (*ω* = 0.812). Moderate values were observed for Punitiveness (*ω* = 0.789), Vulnerability to Harm/Illness (*ω* = 0.774), Insufficient Self‐Control (*ω* = 0.767), Subjugation (*ω* = 0.762), Dependence (*ω* = 0.760), and Enmeshment (*ω* = 0.750). The lowest reliabilities were found for Entitlement/Grandiosity (*ω* = 0.748), Unrelenting Standards (*ω* = 0.732), and Approval‐Seeking (*ω* = 0.726).

### 2.3 Data Analysis

Network analysis (NA) was completed with the software R (R Core Team 2021) in *qgraph* and *bootnet* packages based on Epskamp et al. (2012). Four different NAs were performed for each of the four diagnostic groups: AN, BN, BED, and OB. The networks were estimated with Gaussian Markov random field estimation, with ‘Least Absolute Shrinkage and Selection Operator’ (LASSO) regularization: put a penalty on the correlations closest to zero to drive small correlations automatically towards 0 and retain only strong associations and minimize spurious associations (Albieri and Didelez 2014). The Extended Bayesian Information Criterion (EBIC), a parameter that determines the level of regularization/penalty of sparse correlations, was set to 0.5 in these analyses (values of 0 to 0.5 are typically chosen) (Chen and Chen 2008). Network estimation was calculated with the estimate Network function of the bootnet package (Epskamp et al. 2018). Node centrality was used to approximate node influence, or structural centrality, in the networks. The centrality Plot function of qgraph was used in order to estimate centrality indices. Betweenness (the degree to which a node affects the average path between other pairs of nodes); closeness (the degree to which a node is indirectly linked to the other nodes); strength (the degree to which a node is directly linked to the other nodes) and expected influence (sum of a node's connections as a measure of the relative influence of a node in a network) indices estimate the centrality. Expected influence index was chosen as the main centrality coefficient in consideration of the fact that interpretation of betweenness and closeness in networks is a little ambiguous (Forbes et al. 2017) and the expected influence index is assumed to be a more accurate centrality index than the others (Epskamp and Fried 2018). The correlation stability (CS) coefficient was calculated to check the internal reliability of networks (Epskamp et al. 2018). CS is the largest percentage of populations that can be omitted so that correlation between recalculated indices of resulting networks and indices of the original network is at least 0.7. Minimum cut‐off of 0.25 is recommended for betweenness, closeness and expected influence to consider a network stable (Epskamp et al. 2018). The CS coefficient was approximated using case‐drop bootstrapping (nboots = 2000). Accuracy of edge‐weight was estimated by sampling bootstrapped confidence intervals obtained through nonparametric bootstrapping (nboots = 2000). Analysis of network stability for both case‐drop and nonparametric bootstrapping was carried out by the bootnet function in the *bootnet* package. Visual inspection of the networks reveals the thickness of an edge graphically displaying the strength of the association: more thick edges, more potent associations among symptoms.

## 3 Results

### 3.1 Demographic and Clinical Characteristics

For the AN‐R group, age ranged from 14 to 50 years (M = 19.8, SD = 7.0), with BMI values between 13.1 and 22.0 (M = 17.6, SD = 2.6). In the BN group, age ranged from 14 to 45 years (M = 23.5, SD = 9.2), and BMI ranged from 14.8 to 44.6 (M = 23.8, SD = 5.6). Regarding the BED group, age ranged from 17 to 60 years (M = 37.0, SD = 13.7), with BMI between 21.7 and 61.9 (M = 38.7, SD = 8.5). Finally, the Obesity group showed an age range of 16–61 years (M = 41.9, SD = 12.6) and a BMI range of 30.1–64.7 (M = 42.4, SD = 8.2). As shown in Table 1, women prevailed in all groups, particularly the AN (96.1%) and BN (93.5%) groups, whereas there were relatively more men in the OB group (25.0%). Considering civil status, the majority of AN and BN participants were single, whereas over half of the OB group were married. Educational status revealed most participants had completed high school, with BED and OB groups reporting relatively higher university qualifications. Students represented the largest occupational group in the AN and BN groups, while OB participants were more likely to be unemployed or office workers. Overall, the groups differed extensively in demographic and clinical profiles, which reflected typical developmental and social patterns associated with each diagnosis.

**TABLE 1:** Demographic, clinical, and psychopathological characteristics across eating disorder and obesity groups.

		AN‐R (*N* = 129)		BN (*N* = 124)		BED (*N* = 166)		OB (*N* = 152)	
		Mean	SD	Mean	SD	Mean	SD	Mean	SD
Age		19.8	7.0	23.5	9.2	37.0	13.7	41.9	12.6
Gender^a^	Female	124	(96.1)	116	(93.5)	147	(88.6)	114	(75.0)
	Male	5	(3.9)	8	(6.5)	19	(11.4)	38	(25.0)
BMI		17.6	2.6	23.8	5.6	38.7	8.5	42.4	8.2
Civil status^a^	Single	121	(93.8)	104	(83.9)	76	(45.8)	40	(26.3)
	Married	5	(3.9)	15	(12.1)	76	(45.8)	87	(57.2)
	Cohabiting	3	(2.3)	3	(2.4)	5	(3.0)	9	(5.9)
	Divorced	0	(0.0)	2	(1.6)	8	(4.8)	14	(9.2)
	Widow	0	(0.0)	0	(0.0)	1	(0.6)	2	(1.3)
Education^a^	Primary school	2	(1.6)	2	(1.6)	1	(0.6)	8	(5.3)
	Middle school	67	(51.9)	45	(36.3)	38	(22.9)	46	(30.3)
	High school	52	(40.3)	61	(49.2)	92	(55.4)	72	(47.4)
	University degree	8	(6.2)	16	(12.9)	35	(21.1)	26	(17.1)
Occupation^a^	Housewife	2	(1.6)	6	(4.8)	24	(14.5)	31	(20.4)
	Employee	4	(3.1)	10	(8.1)	25	(15.1)	27	(17.8)
	Unemployed	11	(8.5)	15	(12.1)	24	(14.5)	37	(24.3)
	Office worker	13	(10.1)	13	(10.5)	49	(29.5)	37	(24.3)
	Retired	0	(0.0)	1	(0.8)	4	(2.4)	5	(3.3)
	Student	99	(76.7)	79	(63.7)	40	(24.1)	15	(9.9)
YSQ‐S3	Emotional deprivation	2.3	1.2	2.8	1.2	2.6	1.3	1.8	0.9
	Abandonment	3.0	1.3	3.7	1.4	3.0	1.4	2.0	1.1
	Mistrust/Abuse	3.1	1.3	3.7	1.4	3.0	1.2	2.2	1.1
	Social isolation	2.8	1.3	3.6	1.3	2.9	1.3	1.8	1.0
	Defectiveness/Shame	2.7	1.4	3.3	1.5	2.4	1.3	1.5	0.8
	Failure	2.5	1.4	3.5	1.6	2.8	1.4	1.6	0.9
	Dependence/Incompetence	2.2	0.9	2.9	1.2	2.3	1.0	1.6	0.8
	Vulnerability to harm/Illness	2.1	0.9	2.8	1.4	2.5	1.1	1.7	0.8
	Enmeshment	2.1	0.9	2.7	1.2	2.5	1.1	1.9	0.8
	Subjugation	2.2	1.1	3.0	1.3	2.5	1.2	1.7	0.8
	Self‐sacrifice	3.6	1.2	4.2	1.3	4.0	1.2	3.7	1.2
	Emotional inhibition	2.9	1.2	3.3	1.3	2.6	1.1	2.1	1.0
	Unrelenting standards	3.5	0.9	3.7	1.1	3.3	0.9	2.9	0.8
	Entitlement/Grandiosity	2.6	0.8	2.9	1.0	2.8	1.1	2.4	1.0
	Insufficient self‐control	2.5	1.0	3.5	1.2	3.3	1.2	2.1	0.9
	Approval seeking	3.0	1.1	3.2	1.2	3.2	1.2	2.3	1.0
	Negativity/Pessimism	3.2	1.2	3.8	1.3	3.3	1.3	2.3	1.5
	Punitiveness	2.9	1.2	3.5	1.4	2.7	1.2	2.1	1.2
EDE	Restraint	3.2	2.0	4.1	1.5	2.4	1.7	1.9	1.5
	Eating concern	2.7	1.5	4.0	1.3	3.3	1.5	1.3	1.3
	Shape concern	4.1	1.7	5.1	1.1	4.7	1.4	3.7	1.6
	Weight concern	3.4	1.7	4.7	1.3	4.2	1.4	2.9	1.3
	Total score	3.4	1.6	4.5	1.1	3.7	1.2	2.5	1.2

Abbreviations: AN‐R: anorexia nervosa–restricting type; BED: binge‐eating disorder; BMI: body mass index; BN: bulimia nervosa; EDE: Eating Disorder Examination; OB: obesity; SD: standard deviation; YSQ‐S3: Young Schema Questionnaire Short Form‐3.

^a^ Data are expressed as frequencies and (percentages).

### 3.2 NA in Patients With AN

**FIGURE 1:**
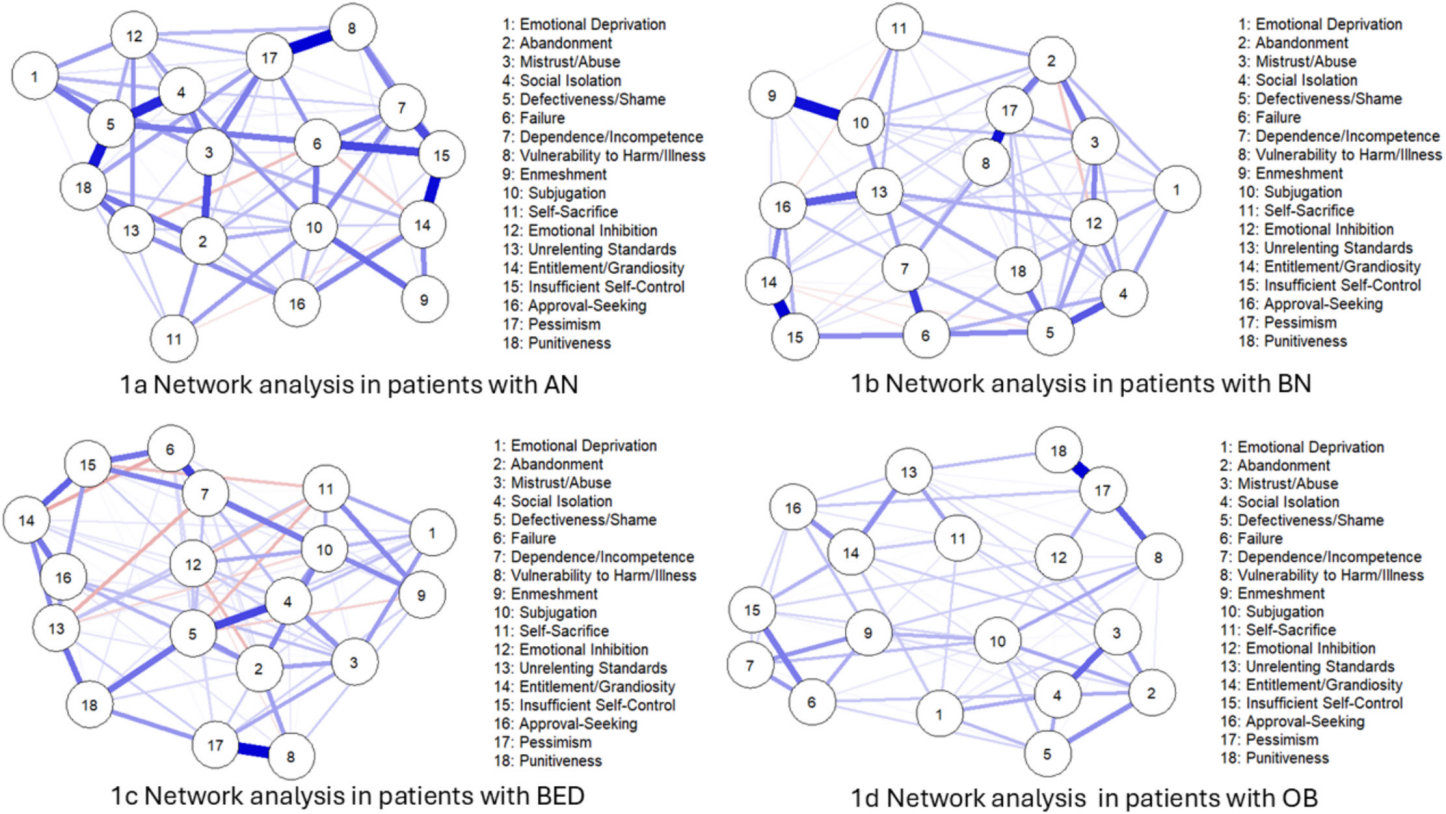
Network structures of early maladaptive schemas (EMSs) in four diagnostic groups: (a) anorexia nervosa (AN), (b) bulimia nervosa (BN), (c) binge‐eating disorder (BED), and (d) obesity (OB). Node proximity and edge thickness represent the strength of partial correlations between EMSs.

The network for patients with AN is represented in Figure 1a. Of the most central of the nodes, in terms of expected influence (EI), were Defectiveness/Shame (EI = 1.24), Negativity/Pessimism (EI = 1.20), and Subjugation (EI = 1.12). At the opposite end of the continuum, Self‐Sacrifice (EI = −2.03) and Enmeshment (EI = −1.86) had the most negative expected influence values and so peripheral locations in the network. Centrality results for strength, betweenness, and closeness are provided in the supplementary materials (Figure S1). The expected influence correlation stability coefficient (CS) was 0.364, considerably greater than the 0.25 threshold for a high interpretability of node centrality. Among the strongest partial correlations in the network (Table S1) were Negativity/Pessimism and Vulnerability to Harm/Illness (*r* = 0.35), Insufficient Self‐Control and Entitlement/Grandiosity (*r* = 0.34), Defectiveness/Shame and Punishment (*r* = 0.32), and Defectiveness/Shame and Social Isolation (*r* = 0.30). These findings point to strong direct links between unfavourable future expectancies, subjective vulnerability, impulsivity, entitlement, and self‐criticisms within the AN group.

Additional files are provided for the accuracy of the CS‐coefficient indices (Figure S2), as well as for bootstrapped confidence intervals for the edge‐weight estimation (Figure S3) and centrality estimation (Figure S4).

### 3.3 NA in Patients With BN

Figure 1b illustrates the network of patients with BN. Node centrality using Expected Influence revealed that the three most central schemas in the network were Subjugation (EI = 1.55), Defectiveness/Shame (EI = 1.30), and Negativity/Pessimism (EI = 1.30). These nodes also presented the highest positive influence ratings, and they demonstrated good direct connectivity with other nodes in the network. Conversely, nodes such as Self‐Sacrifice (EI = −2.01), Enmeshment/Undeveloped Self (EI = −1.50), and Emotional Deprivation (EI = −1.05) had the lowest EI scores, suggesting they are more peripheral and negatively connected in the schema network. Additional centrality indices (strength, betweenness, and closeness) are available in the supplementary materials. (Figure S5).

The CS‐coefficient of expected influence was 0.282, well above the suggested minimum of 0.25, thereby suggesting acceptable stability and interpretability of node centrality estimates in this network.

Among the most significant partial correlations in the network (Table S2) were three significant associations: Entitlement/Grandiosity and Insufficient Self‐Control (*r* = 0.43), Subjugation and Enmeshment/Undeveloped Self (*r* = 0.40), and Negativity/Pessimism and Vulnerability to Harm/Illness (*r* = 0.40). These significant correlations suggest that individuals higher in entitlement or grandiose expectations would also be more likely to be impulsive and have poor self‐discipline. Further, overyielding to others' demands was highly correlated with reduced personal autonomy and enmeshment, while pervasive pessimism concerning the future was highly correlated with feeling vulnerable to outside harm or sickness. These findings emphasize the presence of densely interconnected schema domains in the BN group and indicate possible intervention targets. Refer to Additional files for the CS‐coefficient indices reliability (Figure S6), for the bootstrapped confidence interval of the edge‐weight estimates (Figure S7) and centrality index estimates (Figure S8).

### 3.4 NA in Patients With BED

Figure 1c presents the network for patients with BED. Node centrality analysis using expected influence described some central schemas with leadership roles in the BED network. The most central nodes and the highest positive EI values were Subjugation (EI = 1.52), Negativity/Pessimism (EI = 1.38), Social Isolation (EI = 1.18), and Abandonment (EI = 1.06). These schemas had the strongest direct influence on other nodes in the network, suggesting that they play a central role in propagating the maladaptive schema configuration among individuals with BED. By contrast, nodes such as Self‐Sacrifice (EI = −2.26), Emotional Inhibition (EI = −1.64), and Enmeshment/Undeveloped Self (EI = −1.09) reported the most negative EI scores, suggesting that they are most peripheral but may also represent disengagement or suppression in the schema system. Results for strength, betweenness, and closeness centrality are reported in the supplementary materials (Figure S9).

The CS‐coefficient of expected influence was 0.44, which indicates a very high centrality stability. The value is greater than the recommended 0.25 threshold for interpretability, and it suggests that the rank‐order of EI across nodes remains stable even if up to 44% of the sample is eliminated. This kind of robustness ensures the validity of inferences from node centrality estimates in the network.

Among the highest partial correlations in the BED network (Table S3), three significant correlations were found. There was a close correlation between Negativity/Pessimism and Vulnerability to Harm/Illness (*r* = 0.45), which would suggest that people with a pessimistic outlook are likely to develop severe fears of illness and harm from others. Additionally, a high correlation was established between Failure and Dependence/Incompetence (*r* = 0.33) indicating an intimate association between self‐perceived failure and the experience of helplessness or overdependence on others. Lastly, Social Isolation was also strongly associated with Defectiveness/Shame (*r* = 0.32), indicating the way that social withdrawal can co‐occur with deep self‐blame and worthlessness. These correlations emphasize the interconnected nature of maladaptive schemas in BED and indicate clinically useful targets for intervention.

Additional files include Figure S10, which presents the accuracy of the CS‐coefficient indices, along with Figures S11 and S12, which show bootstrapped confidence intervals for edge‐weight and centrality estimations, respectively.

### 3.5 NA in Patients With OB

Figure 1d illustrates the network of patients with OB. Network centrality node analysis with expected influence indicated several central schemas that were taking key roles in obesity patient networks. The most central of the nodes with the strongest positive EI values were Negativity/Pessimism (EI = 1.81), Social Isolation (EI = 1.53), Subjugation (EI = 1.17), and Mistrust/Abuse (EI = 1.06). These schemas exerted the strongest direct influence on other nodes in the network, suggesting their central place in the maintenance of the maladaptive schema configuration in this clinical group. However, nodes such as Self‐Sacrifice (EI = −1.27), Punitiveness (EI = −1.61), and Emotional Inhibition (EI = −1.13) contained the lowest EI ratings and may therefore represent more peripheral or repressed internal schema processes. Centrality results for strength, betweenness, and closeness are provided in the supplementary materials (Figure S13).

The CS‐coefficient for expected influence was 0.441, which represents a high centrality stability.

Among the strongest partial correlations in the network (Table S4), three associations emerged. The highest correlation was between Negativity/Pessimism and Punitiveness (*r* = 0.54), meaning that there is a strong correlation between pessimism and punitive or self‐critical tendencies. Negativity/Pessimism was also correlated with Vulnerability to Harm/Illness (*r* = 0.34), meaning that individuals who have the tendency to worry and anticipate catastrophes are also more likely to exhibit high levels of fear for physical illness or harm. Finally, a strong relationship was found between Mistrust/Abuse and Social Isolation (*r* = 0.32), which tells us how expectations for interpersonal threat may be followed by social withdrawal or isolation. The results highlight the interconnectedness of network model maladaptive cognitions of pessimism, threat perception, and interpersonal distrust.

Please refer to the additional files for the reliability of the CS‐coefficient indices (Figure S14), as well as the bootstrapped confidence intervals for edge‐weight estimates (Figure S15) and centrality index estimates (Figure S16).

## 4 Discussion

The current study applied network analysis to examine the structure of EMSs among patients with AN, BN, BED, and OB. Among these distinct diagnostic groups, findings indicate transdiagnostic and diagnosis‐specific patterns of schemas that yield considerable insight into the cognitive‐affective structure of eating and weight disorders.

### 4.1 Subjugation and Negativity/Pessimism as Core Transdiagnostic Schemas

Across all diagnostic groups, Negativity/Pessimism and Subjugation were the two most widespread schemas—in line with their potential roles as transdiagnostic cognitive vulnerabilities common across eating disorders (Table 2). This finding is in accordance with recent speculations that emotional resignation and interpersonal surrender may play an important function in driving and maintaining eating disorder behaviours (Simpson and Smith 2019).

**TABLE 2:** Summary of central schemas with their expected influence value, stability coefficients, strongest partial correlations, and transdiagnostic schema common across diagnostic groups.

	AN‐R	BN	BED	Obesity
Central schemas (EI values)	Subjugation (EI = 1.12) Negativity/Pessimism (EI = 1.20) Defectiveness/Shame (EI = 1.24)	Subjugation (EI = 1.55) Negativity/Pessimism (EI = 1.30) Defectiveness/Shame (EI = 1.30)	Subjugation (EI = 1.52) Negativity/Pessimism (EI = 1.38) Social Isolation (EI = 1.18) Abandonment (EI = 1.06)	Subjugation (EI = 1.17) Negativity/Pessimism (EI = 1.81) Social Isolation (EI = 1.53) Mistrust/Abuse (EI = 1.06)
CS‐coefficient for EI	0.364	0.282	0.440	0.441
Strong partial correlation	Negativity/Pessimism and Vulnerability to Harm/Illness (*r* = 0.35) Insufficient Self‐Control and Entitlement/Grandiosity (*r* = 0.34) Defectiveness/Shame and Punishment (*r* = 0.32) Defectiveness/Shame and Social Isolation (*r* = 0.30).	Negativity/Pessimism and Vulnerability to Harm/Illness (*r* = 0.40). Insufficient Self‐Control and Entitlement/Grandiosity (*r* = 0.43) Subjugation and Enmeshment/Undeveloped Self (*r* = 0.40)	Negativity/Pessimism and Vulnerability to Harm/Illness (*r* = 0.45), Failure and Dependence/Incompetence (*r* = 0.33) Social Isolation and Defectiveness/Shame (*r* = 0.32)	Negativity/Pessimism and Vulnerability to Harm/Illness (*r* = 0.34) Negativity/Pessimism and Punitiveness (*r* = 0.54) Mistrust/Abuse and Social Isolation (*r* = 0.32)
Transdiagnostic schemas	Subjugation, Negativity/Pessimism, Defectiveness/Shame			

Abbreviations: AN‐R: anorexia nervosa–restricting type; BED: binge‐eating disorder; BN: bulimia nervosa; CS: correlation stability coefficient; EI: expected influence.

Subjugation, referring to chronic compliance and self‐silencing because of the fear of disagreeing or being rejected, was prominent across all groups. Its presence has been consistently documented in individuals with EDs and OB (Basile et al. 2019; Unoka et al. 2010) and its robust association across diagnostic categories in the present study further underscores its broad clinical relevance and generalizability. The pervasiveness of Subjugation suggests that overcompliance mediated by fear and guilt may be core affective processes across EDs even when manifest external symptomatology is highly variable. While shame has traditionally received more focus in the eating disorder literature (Kenny et al. 2024; Nechita et al. 2021; O'Loghlen et al. 2022), recent perspectives have pointed to guilt as an overlooked yet clinically relevant emotion (Raffone et al. 2025). The current findings support the view that guilt‐related schema processes, such as subjugation, warrant deeper integration into theoretical models and therapeutic approaches.

Negativity/Pessimism, as a global expectation of failure, disappointment, and hardship, was similarly always central. The schema could imply a broader pattern of helplessness and emotional resignation learning, especially observed in BED and OB (Aloi, Rania, Caroleo, et al. 2020; Spirou et al. 2022). Prior research links pessimism to depression and hopelessness in EDs (Mansfield and Wade 2000; Marco et al. 2020; Sonnenblick et al. 2024), and our results affirm its role as a key vulnerability across diagnostic presentations.

Subjugation and Negativity/Pessimism appear as recurring schema constellations, shaping a psychological landscape marked by helpless compliance and emotional disconnection. Subjugation of one's own needs and emotions, alongside lowering expectations in anticipation of disappointment, may function to appease others in order to preserve attachments and prevent activation of underlying Defectiveness/Shame and/or Abandonment schemas. This pattern may help explain the reliance on disordered eating behaviours as indirect strategies for regulating unmet emotional needs, particularly in contexts where self‐assertion feels unsafe or ineffective.

### 4.2 AN and BN: Shame, Surrender, and Emotional Overcontrol

For AN and BN alike, the most central schemas were Defectiveness/Shame, Negativity/Pessimism, and Subjugation, coming together in a shared emotional‐cognitive profile centred on guilt, shame, and emotional inhibition.

This triad suggests an internalized feeling of personal inadequacy (Defectiveness) that is very extreme, coupled with negative future expectations (Pessimism), and a subjugation interpersonal style of communication. While AN is generally associated with overcontrol and perfectionism, and BN with impulsivity and dysregulation, this network equivalence suggests that they have in common a similar underlying experience of basically feeling defective, powerless, and unable to meet one's own needs.

Previous studies have consistently found elevated levels of shame and self‐criticism in AN and BN (Kelly and Tasca 2016; Paranjothy and Wade 2024), and high Subjugation in BN (Elmquist et al. 2015; Leung et al. 1999; Pauwels et al. 2018). Our findings complement and extend this work through the evidence of how such schemas function as interconnected nodes within a wider maladaptive schema network, suggesting that treatment approaches will need to address directly shame, guilt, and repression of emotional needs.

### 4.3 BED and OB: Emotional Disconnection and Interpersonal Withdrawal

In BED and OB, the shared central schemas were Subjugation, Negativity/Pessimism, and Social Isolation, and these are another but similarly maladaptive schema constellation. These people appear to view the world from a perspective of interpersonal isolation, emotional hopelessness, and self‐denial, which may lead them to binge eat or overeat as coping strategies for unidentified emotional pain and unmet relational needs.

The strong role of *Social Isolation* is consistent with previous literature and qualitative findings. Individuals with BED and obesity often report profound feelings of loneliness, deficits in social support, and pervasive interpersonal mistrust (Brugnera et al. 2018; Jung and Luck‐Sikorski 2019).

Waller further emphasizes that, while core beliefs were not significantly associated with bulimic behaviours in individuals with bulimia nervosa, *social isolation* was significantly and positively associated with binge‐eating behaviours in those with BED—highlighting a disorder‐specific role of this schema in the severity of pathology (Waller 2003). The prominence of the *Social Isolation* schema in BED and obesity may also reflect early adverse experiences such as weight stigma, bullying, and social exclusion, which contribute to entrenched beliefs of nonbelonging and rejection (Albano et al. 2019; Aloi, Rania, Caroleo, et al. 2020; Hollett and Carter 2021). In the framework of schema therapy, this pattern may indicate not only withdrawal but also resignation to emotional abandonment, aligning with the conceptualization of avoidance and surrender coping modes (Young et al. 2003).

Significantly, while both BED and OB shared this core profile, they differed on one key schema. Of particular note, Abandonment was also significant in the BED network. This is consistent with research that patients with BED are prone to extreme fear of rejection and loss, and may use food for self‐soothing or as a replacement for deficient attachments (Aloi, Rania, Caroleo, et al. 2020; Maxwell et al. 2017). This result, in addition to the strong correlation between Failure and Dependence/Incompetence, highlights the importance of attachment‐based treatment in BED populations in order to target and improve the domain of Impaired Autonomy and Performance, which has been found to be associated with BED (Rania et al. 2020).

On the other hand, Mistrust/Abuse was a central node in the OB network. This suggests that individuals with obesity may anticipate being hurt, taken advantage of, or humiliated by others.

This result is not surprising; in fact, literature reported that adult obesity is related to emotional abuse and neglect (Amianto et al. 2018; Hemmingsson et al. 2014).

This schema may underlie emotional distancing, protective behaviour, and barriers to health care or social support seeking, consistent with recent schema work in obesity (Basile et al. 2019; Spirou et al. 2022).

These distinctions underscore the necessity of adapting schema‐based treatment to the dominant interpersonal themes within each diagnostic group—fear of abandonment (BED) versus suspicion of others' intentions (OB).

### 4.4 Clinical Implications

Our findings suggest both shared therapeutic targets and diagnosis‐specific considerations that can meaningfully inform schema‐based treatment approaches for eating disorders and obesity. Across all ED subtypes and in OB, clinical interventions should consistently address the schemas of Subjugation and Negativity/Pessimism, and the loneliness that follows from these, as a result of never truly being seen or understood by others. These may be targeted through the development of assertiveness skills, the challenging of hopeless or fatalistic cognitions, the exploration of unmet emotional needs, and therapeutic work focused on alleviating excessive guilt.

In AN and BN patients, particular attention should also be placed on the Defectiveness/Shame schema. Targeting this schema is crucial to establish more adaptive self‐structures and reduce the strict internal standards that often maintain disordered eating behaviours in these groups.

For those with BED, the chronic triggering of Abandonment and Social Isolation emphasizes the need for treatments that reinforce secure attachment, develop interpersonal trust, and provide ongoing affective validation. These treatment goals are central to helping people reduce emotional reliance on food and develop more adaptive relational coping styles.

In the case of OB, successful schema therapy would also target the schema of Mistrust/Abuse. The establishment of a sense of interpersonal safety and the reduction of avoidance of nurturant relationships can be particularly beneficial in this group, given the often complicated relational histories and distrust of others that may underpin eating disorder symptoms.

Of particular interest is that the schema constellations in our ED and OB samples—most particularly the centrality of Subjugation, Defectiveness/Shame, Negativity/Pessimism, and Social Isolation—are all strikingly congruent with those frequently uncovered in patients with Avoidant Personality Disorder (AvPD). A recent systematic review and meta‐analysis conducted by Panagiotopoulos and colleagues highlighted a pervasive EMS pattern in AvPD patients with high prevalence rates of emotional inhibition, social isolation, defectiveness, and subjugation (Panagiotopoulos et al. 2023). These schemas reflect early interpersonal situations of emotional invalidation and guilt‐inducing or controlling interpersonal states, to encourage patterns of interpersonal withdrawal, self‐silencing, and expectations of nonfulfilment of emotional needs. Convergence of ED/OB and AvPD schema profiles suggests that schema therapy can be a unifying transdiagnostic model of understanding and treatment of co‐morbid or phenotypically overlapping emotional and interpersonal distress. This overlap accords with the theory that some presentations of EDs should be understood, in part, as expressions of maladaptive personality styles based on similar schema processes (Aloi et al. 2025).

Taken together, these schema constellations provide a framework for more nuanced and mode‐sensitive interventions. Rather than focusing solely on surface‐level symptoms, they encourage a deeper therapeutic engagement with the core self‐other representations that drive and maintain eating disorder behaviours. This person‐centred approach may ultimately support more sustainable clinical change across diagnostic boundaries.

### 4.5 Strengths, Limitations, and Future Directions

Strengths of this study are a large, diagnostically diverse clinical sample, tight network estimation procedures, and mutual fertilization of initial schema theory with modern statistical modelling. Importantly, this is also one of the few studies conducted on a clinical population, which enhances the clinical relevance of the findings. Moreover, the results proved to be sufficiently robust across diagnostic groups, further strengthening the validity of the conclusions.

Weaknesses are cross‐sectional design and employment of self‐report measures that do not assess schema modes or real‐time affect coping. Another limitation is that participants were recruited from a single specialized clinical centre, and this may introduce selection bias and restrict generalizability of findings. Furthermore, the inclusion of underage patients (< 18 years) reflects the actual clinical population of our centre, where adolescents and adults are treated concurrently; however, this also introduces developmental heterogeneity that must be controlled for when considering the results. In addition, we did not comprehensively measure psychiatric comorbidities; while this choice allowed us to focus schema structure within ED and obesity groups, it prevents consideration of the capacity to contextualize how EMSs interface with extended clinical presentations.

Finally, longitudinal trends of schema centrality and how these evolve over the course of treatment, and to clarify active ingredients of therapeutic change, need to be explored in future studies. The application of ecological momentary assessment (EMA) methodology has the potential to offer more insight into the activation of specific schemas, such as Subjugation and Negativity/Pessimism, in dynamic daily experience. In this vein, trials must evaluate whether direct targeting of the schemas enhances treatment outcomes across diagnoses, and that may be useful in transdiagnostic treatment and in schema‐focused treatments.

## 5 Conclusion

The current study provides strong support that Subjugation and Negativity/Pessimism are transdiagnostic schemata that underlie AN, BN, BED, and OB. Certain constellations—Shame and Guilt‐focused in AN/BN, and interpersonal alienation in BED/OB—are also more effectively illuminating schema‐driven processes in EDs. Such evidence demands the integration of schema therapy as a flexible, tailor‐made treatment, with the possibility of addressing both shared and unique psychological vulnerabilities among patients with EDs and obesity.

## Author Contributions

**Matteo Aloi:** conceptualization; data curation; methodology; writing – original draft; writing – review and editing. **Marianna Rania:** data curation; writing – review and editing. **Elvira Anna Carbone:** data curation; writing—review and editing. **Renato de Filippis:** data curation; writing – review and editing. **Ettore D'Onofrio:** data curation. **Lavinia Rotella:** data curation. **Daria Quirino:** data curation. **Susan Simpson:** supervision; writing – review and editing. **Cristina Segura Garcia:** supervision; writing – review and editing.

## Ethics Statement

The study followed all relevant ethical guidelines. All procedures performed were in accordance with the ethical standards of the institutional research committee and with the 1964 Helsinki Declaration and its later amendments (or comparable ethical standards).

## Consent

Informed consent has been obtained from all participants, and the investigation has been conducted according to the best principles of research with human beings.

## Conflicts of Interest

The authors declare no conflicts of interest.

## Supporting information


**Figure S1:**Centrality indices of early maladaptive schemas in the Anorexia Nervosa patient network estimated with EBICglasso. Strength, betweenness, closeness, and expected influence are reported for each schema.
**Figure S2:**. Results of case‐dropping subset bootstrap procedure to assess stability of network centrality indices. Average correlations between centrality indices of networks sampled with persons dropped and the original sample of person with Anorexia Nervosa. Lines indicate the means and areas indicate the range from the 2.5^th^ quantile to the 97.5^th^ quantile.
**Figure S3:**. Bootstrapped confidence intervals (#boots = 2000) for estimated edge‐weights of network analysis in the Anorexia Nervosa group.
**Figure S4:** Bootstrapped confidence intervals (#boots = 2000) for estimated centrality indices of network analysis in the Anorexia Nervosa group.
**Figure S5:**. Centrality indices of early maladaptive schemas in the Bulimia Nervosa patient network estimated with EBICglasso. Strength, betweenness, closeness, and expected influence are reported for each schema.
**Figure S6:**. Results of case‐dropping subset bootstrap procedure to assess stability of network centrality indices. Average correlations between centrality indices of networks sampled with persons dropped and the original sample of person with Bulimia Nervosa. Lines indicate the means and areas indicate the range from the 2.5^th^ quantile to the 97.5^th^ quantile.
**Figure S7:** Bootstrapped confidence intervals (#boots = 2000) for estimated edge‐weights of network analysis in the Bulimia Nervosa group.
**Figure S8:** Bootstrapped confidence intervals (#boots = 2000) for estimated centrality indices of network analysis in the Bulimia Nervosa group.
**Figure S9:**. Centrality indices of early maladaptive schemas in the Binge‐eating disorder patient network estimated with EBICglasso. Strength, betweenness, closeness, and expected influence are reported for each schema.
**Figure S10:**. Results of case‐dropping subset bootstrap procedure to assess stability of network centrality indices. Average correlations between centrality indices of networks sampled with persons dropped and the original sample of person with Binge‐eating disorder. Lines indicate the means and areas indicate the range from the 2.5^th^ quantile to the 97.5^th^ quantile.
**Figure S11:**. Bootstrapped confidence intervals (#boots = 2000) for estimated edge‐weights of network analysis in the Binge‐eating disorder group.
**Figure S12:** Bootstrapped confidence intervals (#boots = 2000) for estimated centrality indices of network analysis in the Binge‐eating disorder group.
**Figure S12:** Bootstrapped confidence intervals (#boots = 2000) for estimated centrality indices of network analysis in the Binge‐eating disorder group.
**Figure S13:**. Centrality indices of early maladaptive schemas in the Obesity patient network estimated with EBICglasso. Strength, betweenness, closeness, and expected influence are reported for each schema.
**Figure S14:**. Results of case‐dropping subset bootstrap procedure to assess stability of network centrality indices. Average correlations between centrality indices of networks sampled with persons dropped and the original sample of person with Obesity. Lines indicate the means and areas indicate the range from the 2.5^th^ quantile to the 97.5^th^ quantile.
**Figure S15:** Bootstrapped confidence intervals (#boots = 2000) for estimated edge‐weights of network analysis in the Obesity group.
**Figure S16:** Bootstrapped confidence intervals (#boots = 2000) for estimated centrality indices of network analysis in the Obesity group.

**Table S1:** Partial correlation matrix from network analysis of early maladaptive schemas in patients with anorexia nervosa.
**Table S2:** Partial correlation matrix from network analysis of early maladaptive schemas in patients with bulimia nervosa.
**Table S3:** Partial correlation matrix from network analysis of early maladaptive schemas in patients with binge eating disorder.
**Table S4:** Partial correlation matrix from network analysis of early maladaptive schemas in patients with obesity.

## Data Availability

The data that support the findings of this study are available from the corresponding author upon reasonable request.
