# Supplementary material for: Early Maladaptive Schemas as Core Therapeutic Targets in Eating Disorders and Obesity: A Schema Therapy–Informed Network Analysis
Source: Clin Psychol Psychother. 2025 Sep 22;32(5):e70153. doi: 10.1002/cpp.70153 (PMC12451857; doi:10.1002/cpp.70153)
Supplement: Supplementary file 1 — Figure S1:Centrality indices of early maladaptive schemas in the Anorexia Nervosa patient network estimated with EBICglasso. Strength, betweenness, closeness, and expected influence are reported for each schema. Figure S2:. Results of case‐dropping subset bootstrap procedure to assess stability of network centrality indices. Average correlations between centrality indices of networks sampled with persons dropped and the original sample of person with Anorexia Nervosa. Lines indicate the means and areas indicate the range from the 2.5th quantile to the 97.5th quantile. Figure S3:. Bootstrapped confidence intervals (#boots = 2000) for estimated edge‐weights of network analysis in the Anorexia Nervosa group. Figure S4: Bootstrapped confidence intervals (#boots = 2000) for estimated centrality indices of network analysis in the Anorexia Nervosa group. Figure S5:. Centrality indices of early maladaptive schemas in the Bulimia Nervosa patient network estimated with EBICglasso. Strength, betweenness, closeness, and expected influence are reported for each schema. Figure S6:. Results of case‐dropping subset bootstrap procedure to assess stability of network centrality indices. Average correlations between centrality indices of networks sampled with persons dropped and the original sample of person with Bulimia Nervosa. Lines indicate the means and areas indicate the range from the 2.5th quantile to the 97.5th quantile. Figure S7: Bootstrapped confidence intervals (#boots = 2000) for estimated edge‐weights of network analysis in the Bulimia Nervosa group. Figure S8: Bootstrapped confidence intervals (#boots = 2000) for estimated centrality indices of network analysis in the Bulimia Nervosa group. Figure S9:. Centrality indices of early maladaptive schemas in the Binge‐eating disorder patient network estimated with EBICglasso. Strength, betweenness, closeness, and expected influence are reported for each schema. Figure S10:. Results of case‐dropping subset bootstrap procedure [file CPP-32-e70153-s002.docx]

**Supplementary figures**

| **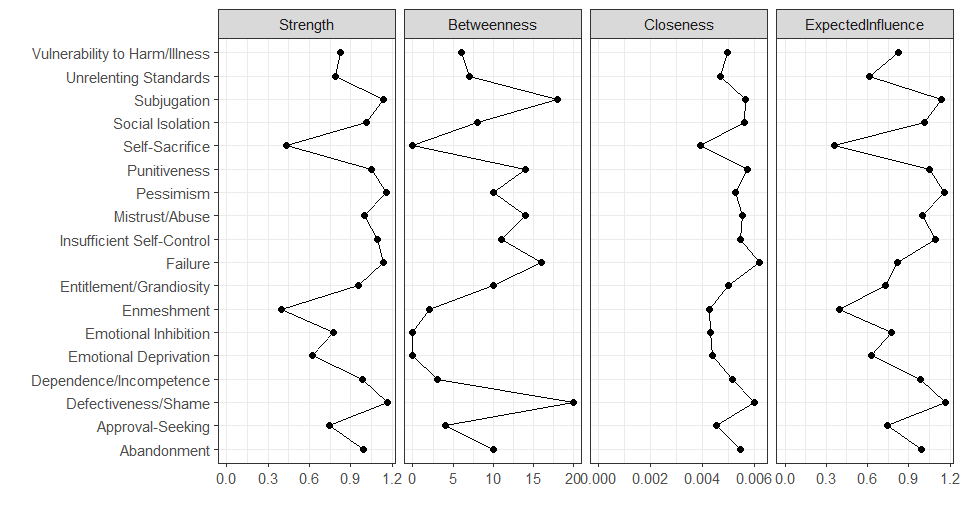** |
| --- |
| ***Figure S1****. Centrality indices of early maladaptive schemas in the Anorexia Nervosa patient network estimated with EBICglasso. Strength, betweenness, closeness, and expected influence are reported for each schema.* |

| 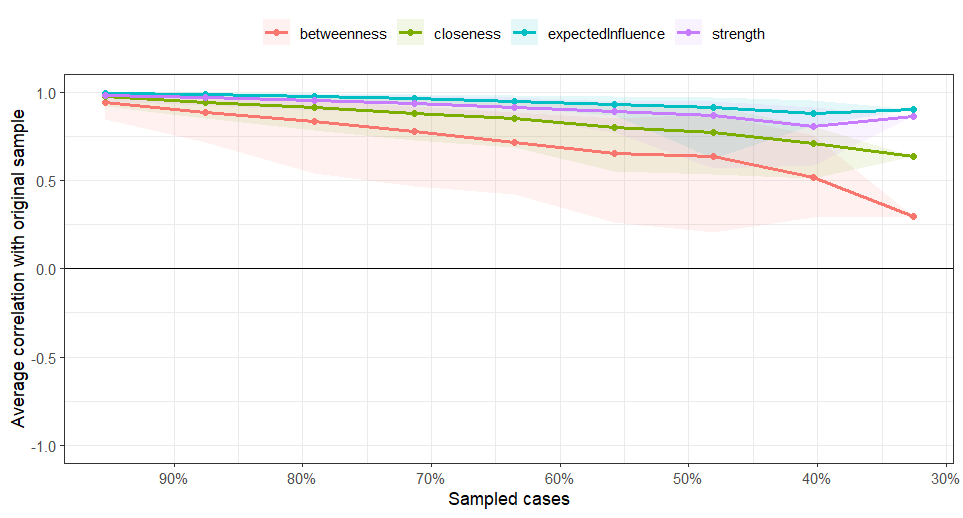 |
| --- |
| ***Figure S2****. Results of case-dropping subset bootstrap procedure to assess stability of network centrality indices. Average correlations between centrality indices of networks sampled with persons dropped and the original sample of person with Anorexia Nervosa. Lines indicate the means and areas indicate the range from the 2.5^th^ quantile to the 97.5^th^ quantile.* |

| 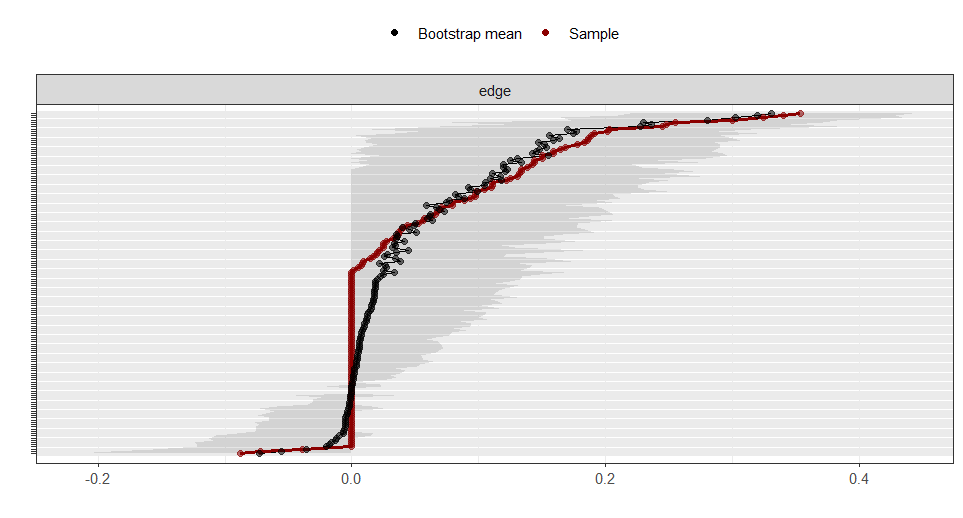 |
| --- |
| ***Figure S3****. Bootstrapped confidence intervals (#boots = 2000) for estimated edge-weights of network analysis in the Anorexia Nervosa group.* |

| 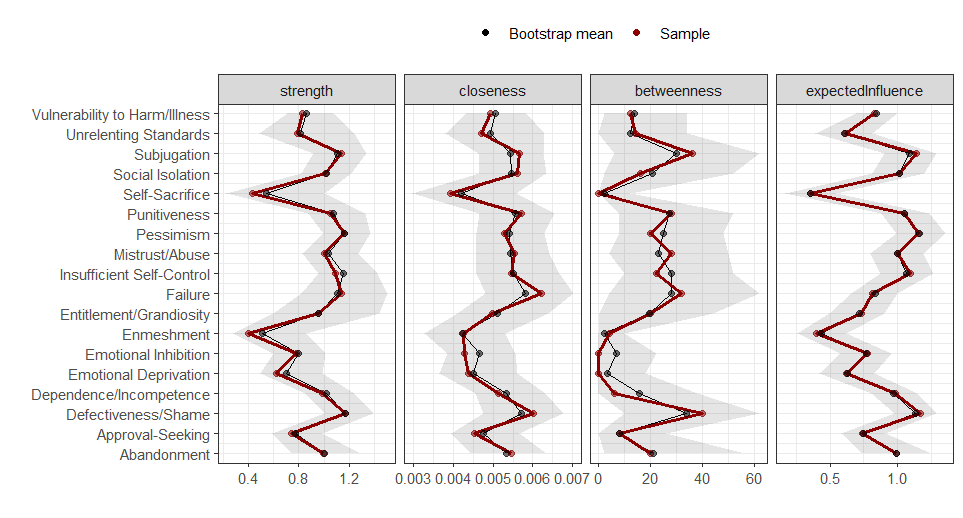 |
| --- |
| *Figure S4. Bootstrapped confidence intervals (#boots = 2000) for estimated centrality indices of network analysis in the Anorexia Nervosa group.* |

| 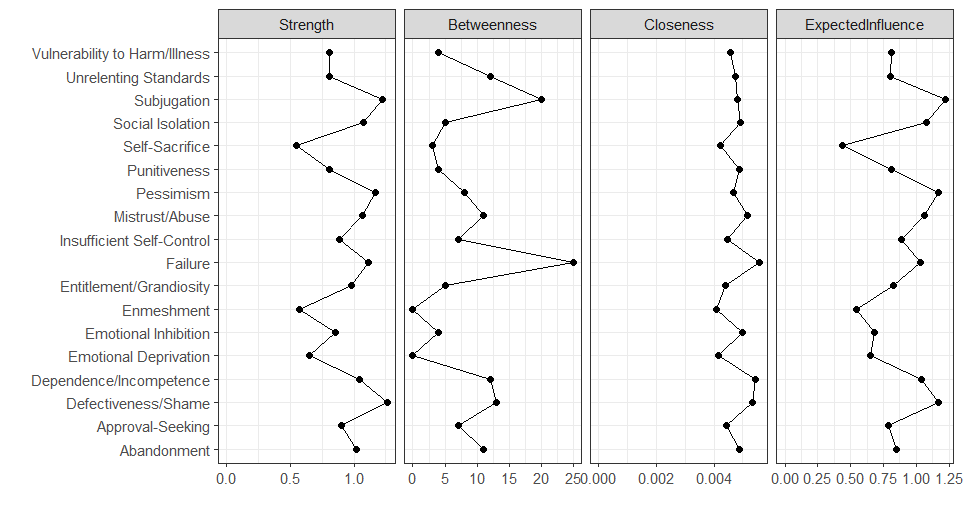 |
| --- |
| ***Figure S5****. Centrality indices of early maladaptive schemas in the Bulimia Nervosa patient network estimated with EBICglasso. Strength, betweenness, closeness, and expected influence are reported for each schema.* |

| 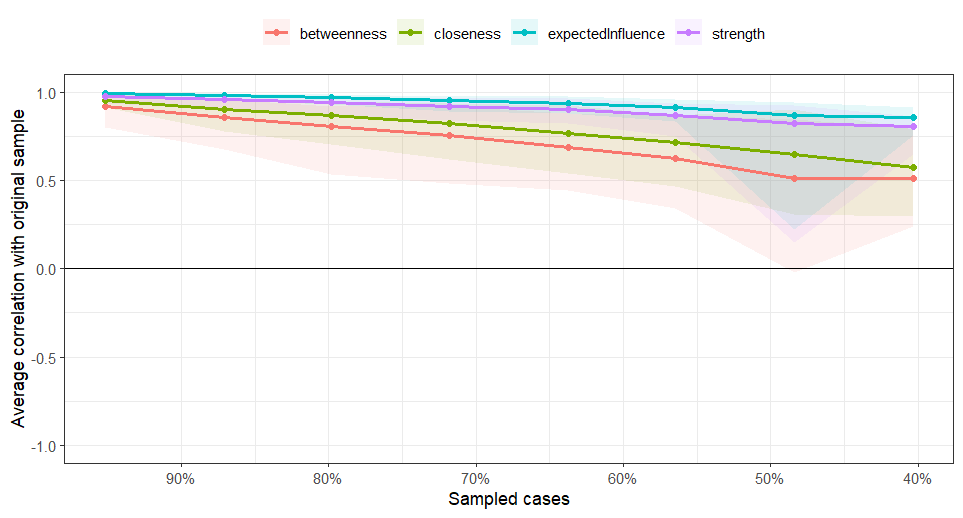 |
| --- |
| ***Figure S6****. Results of case-dropping subset bootstrap procedure to assess stability of network centrality indices. Average correlations between centrality indices of networks sampled with persons dropped and the original sample of person with Bulimia Nervosa. Lines indicate the means and areas indicate the range from the 2.5^th^ quantile to the 97.5^th^ quantile.* |

| 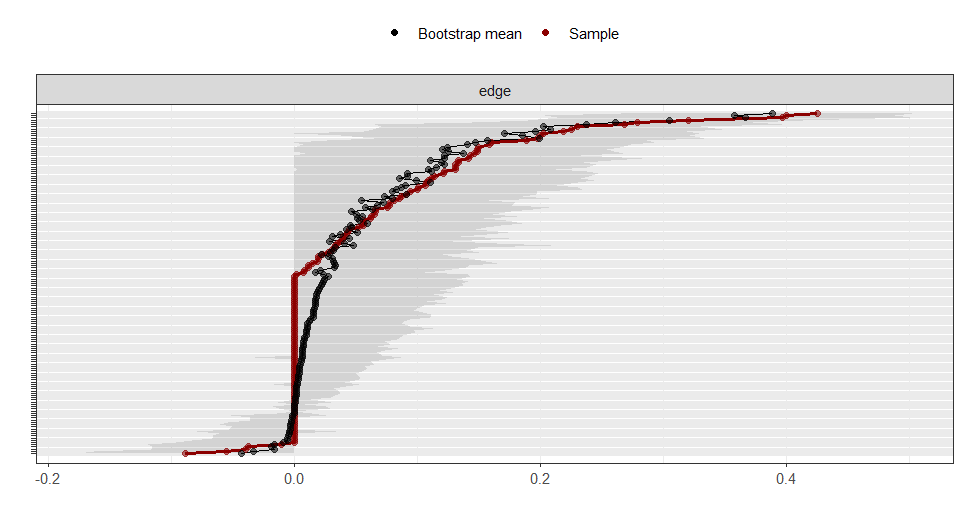 |
| --- |
| ***Figure S7.*** *Bootstrapped confidence intervals (#boots = 2000) for estimated edge-weights of network analysis in the Bulimia Nervosa group.* |

| 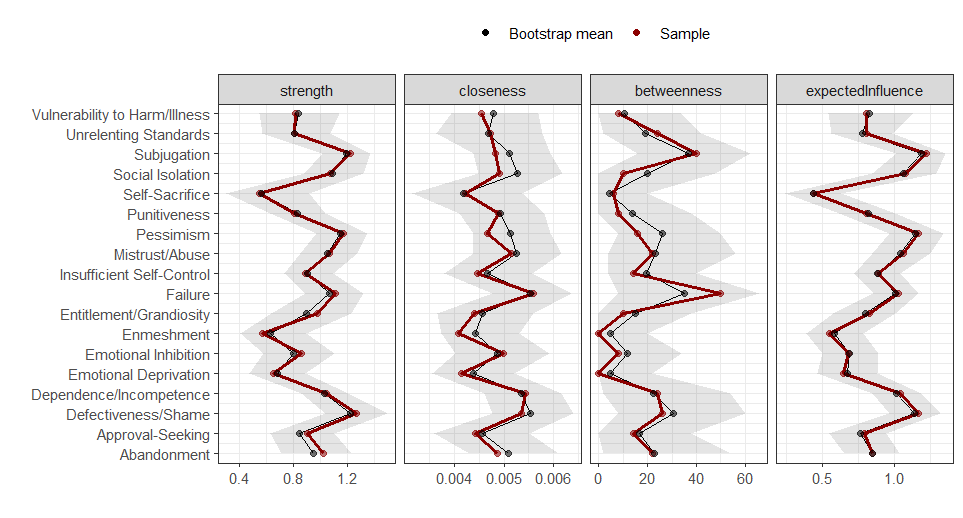 |
| --- |
| ***Figure S8.*** *Bootstrapped confidence intervals (#boots = 2000) for estimated centrality indices of network analysis in the Bulimia Nervosa group.* |

| **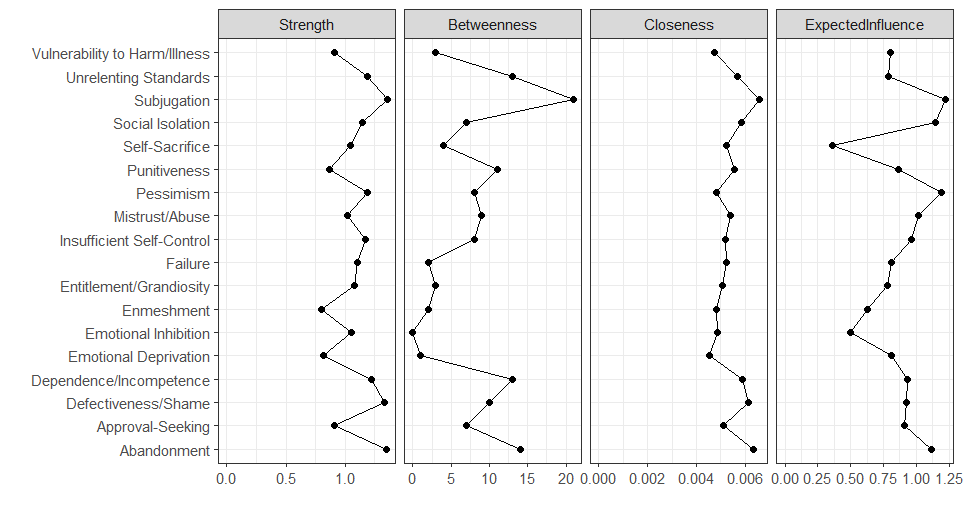** |
| --- |
| ***Figure S9****. Centrality indices of early maladaptive schemas in the Binge-eating disorder patient network estimated with EBICglasso. Strength, betweenness, closeness, and expected influence are reported for each schema.* |

| 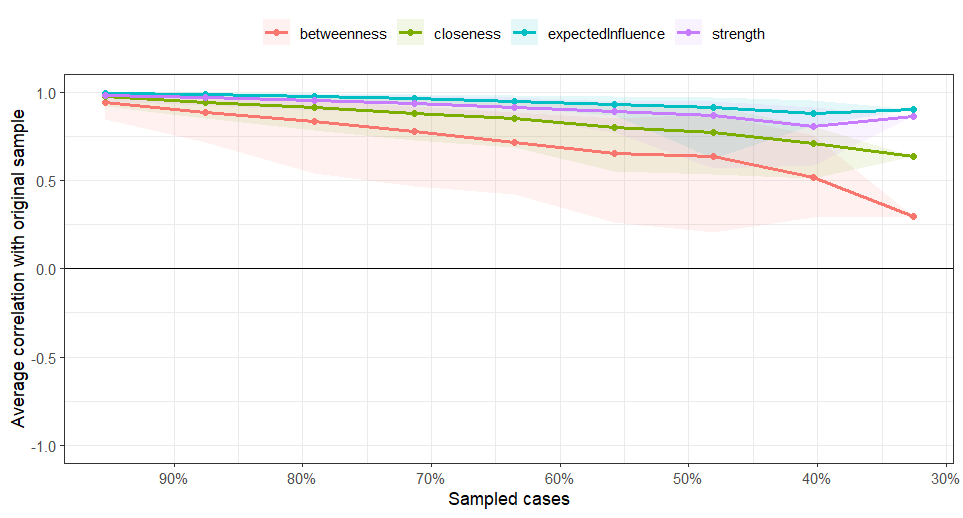 |
| --- |
| ***Figure S10****. Results of case-dropping subset bootstrap procedure to assess stability of network centrality indices. Average correlations between centrality indices of networks sampled with persons dropped and the original sample of person with Binge-eating disorder. Lines indicate the means and areas indicate the range from the 2.5^th^ quantile to the 97.5^th^ quantile.* |

| 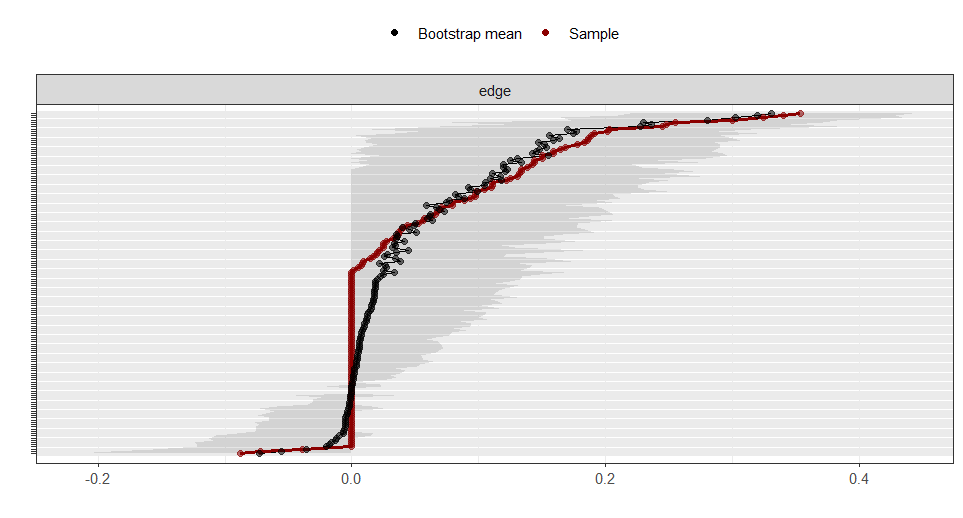 |
| --- |
| ***Figure S11****. Bootstrapped confidence intervals (#boots = 2000) for estimated edge-weights of network analysis in the Binge-eating disorder group.* |

| 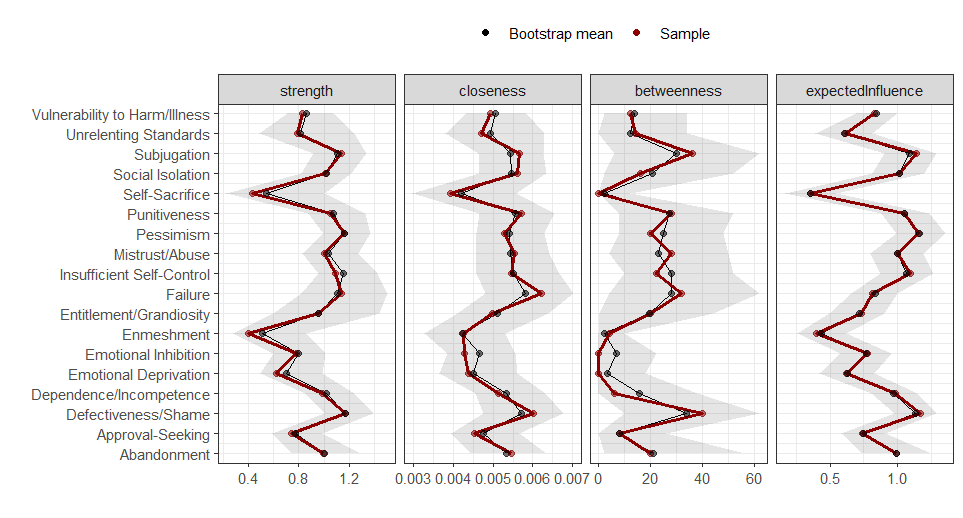 |
| --- |
| *Figure S12. Bootstrapped confidence intervals (#boots = 2000) for estimated centrality indices of network analysis in the Binge-eating disorder group.* |

| 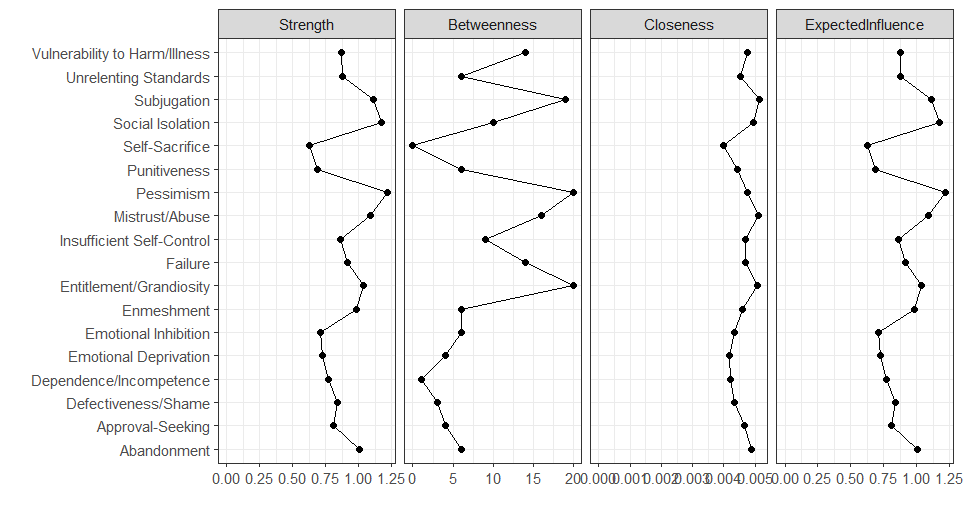 |
| --- |
| ***Figure S13****. Centrality indices of early maladaptive schemas in the Obesity patient network estimated with EBICglasso. Strength, betweenness, closeness, and expected influence are reported for each schema.* |

| 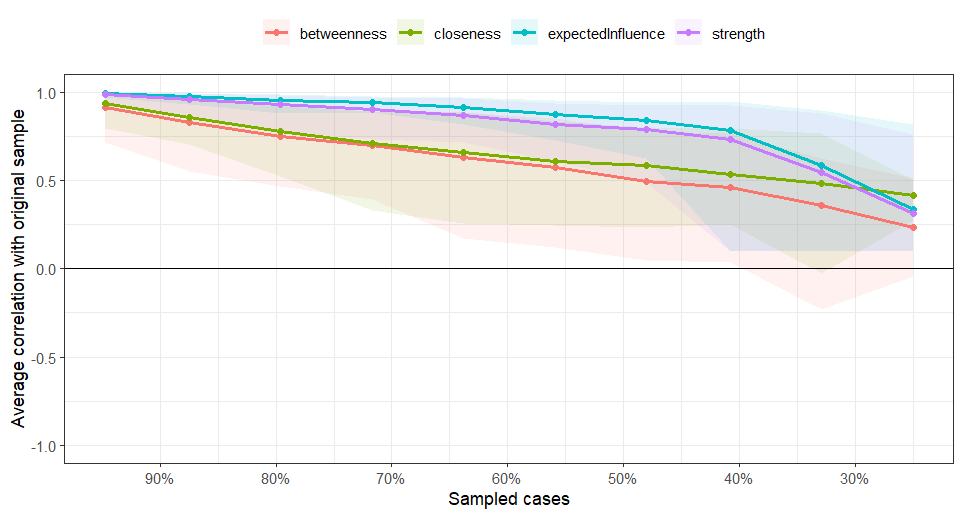 |
| --- |
| ***Figure S14****. Results of case-dropping subset bootstrap procedure to assess stability of network centrality indices. Average correlations between centrality indices of networks sampled with persons dropped and the original sample of person with Obesity. Lines indicate the means and areas indicate the range from the 2.5^th^ quantile to the 97.5^th^ quantile.* |

| 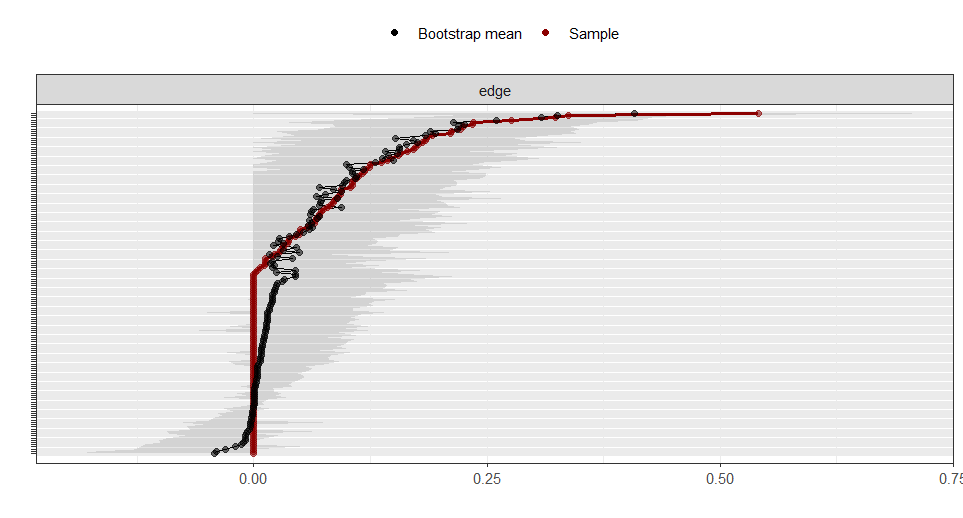 |
| --- |
| ***Figure S15.*** *Bootstrapped confidence intervals (#boots = 2000) for estimated edge-weights of network analysis in the Obesity group.* |

| 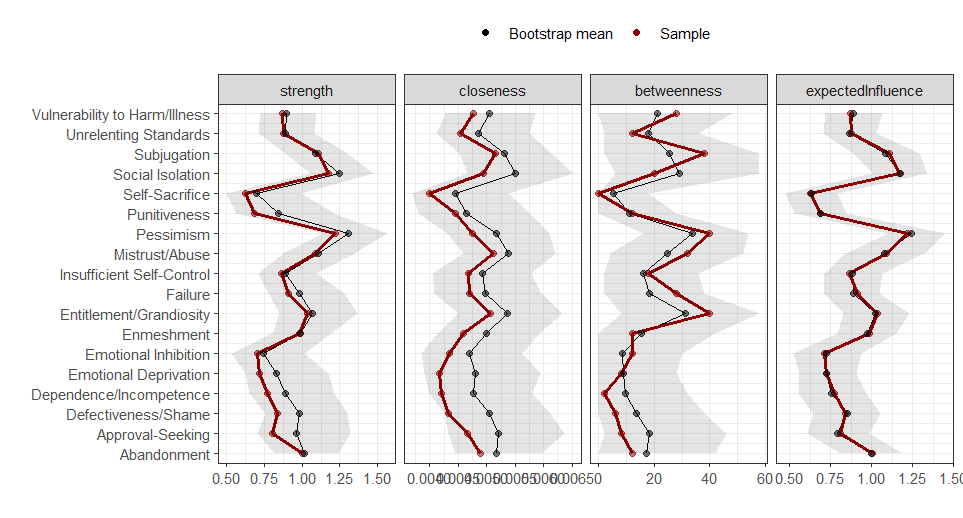 |
| --- |
| ***Figure S16.*** *Bootstrapped confidence intervals (#boots = 2000) for estimated centrality indices of network analysis in the Obesity group.* |
