# Supplementary material for: Early Maladaptive Schemas as Core Therapeutic Targets in Eating Disorders and Obesity: A Schema Therapy–Informed Network Analysis
Source: Clin Psychol Psychother. 2025 Sep 22;32(5):e70153. doi: 10.1002/cpp.70153 (PMC12451857; doi:10.1002/cpp.70153)
Supplement: Supplementary file 2 — Table S1: Partial correlation matrix from network analysis of early maladaptive schemas in patients with anorexia nervosa. Table S2: Partial correlation matrix from network analysis of early maladaptive schemas in patients with bulimia nervosa. Table S3: Partial correlation matrix from network analysis of early maladaptive schemas in patients with binge eating disorder. Table S4: Partial correlation matrix from network analysis of early maladaptive schemas in patients with obesity. [file CPP-32-e70153-s001.docx]

Table S1. Partial correlation matrix from network analysis of early maladaptive schemas in patients with anorexia nervosa.

|  | 1 | 2 | 3 | 4 | 5 | 6 | 7 | 8 | 9 | 10 | 11 | 12 | 13 | 14 | 15 | 16 | 17 | 18 |
| --- | --- | --- | --- | --- | --- | --- | --- | --- | --- | --- | --- | --- | --- | --- | --- | --- | --- | --- |
| 1. Emotional deprivation | - |  |  |  |  |  |  |  |  |  |  |  |  |  |  |  |  |  |
| 2. Abandonment | 0 | - |  |  |  |  |  |  |  |  |  |  |  |  |  |  |  |  |
| 3. Mistrust/Abuse | 0.13 | 0.25 | - |  |  |  |  |  |  |  |  |  |  |  |  |  |  |  |
| 4. Social isolation | 0.1 | 0 | 0.19 | - |  |  |  |  |  |  |  |  |  |  |  |  |  |  |
| 5. Defectiveness/Shame | 0.19 | 0.02 | 0.01 | 0.3 | - |  |  |  |  |  |  |  |  |  |  |  |  |  |
| 6. Failure | 0.01 | 0.04 | 0 | 0 | 0.18 | - |  |  |  |  |  |  |  |  |  |  |  |  |
| 7. Dependence/Incompetence | 0 | 0.09 | 0 | 0.07 | 0.02 | 0.15 | - |  |  |  |  |  |  |  |  |  |  |  |
| 8. Vulnerability to harm/Illness | 0 | 0 | 0 | 0.03 | 0 | 0 | 0.17 | - |  |  |  |  |  |  |  |  |  |  |
| 9. Enmeshment | 0 | 0 | 0 | 0 | 0 | 0 | 0 | 0.04 | - |  |  |  |  |  |  |  |  |  |
| 10. Subjugation | 0.01 | 0.11 | 0 | 0.14 | 0.01 | 0.19 | 0.16 | 0 | 0.2 | - |  |  |  |  |  |  |  |  |
| 11. Self-Sacrifice | 0 | 0.13 | 0 | 0 | 0 | 0 | 0 | 0 | 0 | 0.13 | - |  |  |  |  |  |  |  |
| 12. Emotional inhibition | 0.13 | 0 | 0.08 | 0.12 | 0.11 | 0 | 0 | 0.1 | 0 | 0 | 0 | - |  |  |  |  |  |  |
| 13. Unrelenting standards | 0 | 0 | 0 | 0 | 0 | -0.09 | 0 | 0 | 0 | 0 | 0.07 | 0.14 | - |  |  |  |  |  |
| 14. Entitlement/Grandiosity | 0 | 0 | 0.1 | 0 | 0 | -0.07 | 0 | 0 | 0.16 | 0 | -0.04 | 0 | 0.07 | - |  |  |  |  |
| 15. Insufficient self-control | 0 | 0 | 0.04 | 0.02 | 0 | 0.26 | 0.25 | 0.14 | 0 | 0 | 0 | 0 | 0 | 0.34 | - |  |  |  |
| 16. Approval seeking | 0 | 0.11 | 0.04 | 0 | 0 | 0 | 0.05 | 0 | 0 | 0.1 | 0 | 0 | 0.15 | 0.16 | 0.06 | - |  |  |
| 17. Negativity/Pessimism | 0.04 | 0.06 | 0.18 | 0.01 | 0 | 0.14 | 0.02 | 0.35 | 0 | 0 | 0 | 0.09 | 0.07 | 0.02 | 0 | 0.04 | - |  |
| 18. Punitiveness | 0.02 | 0.19 | 0 | 0.03 | 0.32 | 0 | 0 | 0 | 0 | 0.08 | 0.06 | 0 | 0.2 | 0 | 0 | 0.03 | 0.12 | - |

Table S2. Partial correlation matrix from network analysis of early maladaptive schemas in patients with bulimia nervosa.

|  | 1 | 2 | 3 | 4 | 5 | 6 | 7 | 8 | 9 | 10 | 11 | 12 | 13 | 14 | 15 | 16 | 17 | 18 |
| --- | --- | --- | --- | --- | --- | --- | --- | --- | --- | --- | --- | --- | --- | --- | --- | --- | --- | --- |
| 1. Emotional deprivation | - |  |  |  |  |  |  |  |  |  |  |  |  |  |  |  |  |  |
| 2. Abandonment | 0.11 | - |  |  |  |  |  |  |  |  |  |  |  |  |  |  |  |  |
| 3. Mistrust/Abuse | 0.09 | 0.23 | - |  |  |  |  |  |  |  |  |  |  |  |  |  |  |  |
| 4. Social isolation | 0.15 | 0 | 0.11 | - |  |  |  |  |  |  |  |  |  |  |  |  |  |  |
| 5. Defectiveness/Shame | 0.04 | 0 | 0.09 | 0.28 | - |  |  |  |  |  |  |  |  |  |  |  |  |  |
| 6. Failure | 0 | 0 | 0 | 0.13 | 0.2 | - |  |  |  |  |  |  |  |  |  |  |  |  |
| 7. Dependence/Incompetence | 0 | 0.08 | 0 | 0.01 | 0.13 | 0.32 | - |  |  |  |  |  |  |  |  |  |  |  |
| 8. Vulnerability to harm/Illness | 0 | 0 | 0 | 0.09 | 0.01 | 0 | 0.14 | - |  |  |  |  |  |  |  |  |  |  |
| 9. Enmeshment | 0 | 0 | 0 | 0 | -0.01 | 0 | 0.05 | 0 | - |  |  |  |  |  |  |  |  |  |
| 10. Subjugation | 0.03 | 0.11 | 0.11 | 0.06 | 0 | 0.13 | 0.15 | 0.05 | 0.4 | - |  |  |  |  |  |  |  |  |
| 11. Self-Sacrifice | 0 | 0.13 | 0 | 0 | 0 | 0 | 0 | 0 | 0.02 | 0.15 | - |  |  |  |  |  |  |  |
| 12. Emotional inhibition | 0 | -0.09 | 0.2 | 0.16 | 0.16 | 0 | 0 | 0.06 | 0 | 0 | 0 | - |  |  |  |  |  |  |
| 13. Unrelenting standards | 0 | 0 | 0 | 0 | 0 | 0 | 0 | 0 | 0 | 0 | 0.11 | 0.14 | - |  |  |  |  |  |
| 14. Entitlement/Grandiosity | 0 | 0 | 0.08 | 0.02 | -0.04 | -0.04 | 0 | 0 | 0.07 | 0 | 0 | 0 | 0.07 | - |  |  |  |  |
| 15. Insufficient self-control | 0.01 | 0 | 0 | 0.01 | 0 | 0.19 | 0.06 | 0.04 | 0 | 0 | 0 | 0.04 | 0 | 0.43 | - |  |  |  |
| 16. Approval seeking | 0.08 | 0.06 | 0 | 0 | 0 | 0 | 0.09 | 0 | 0.03 | 0 | -0.06 | 0 | 0.27 | 0.2 | 0.12 | - |  |  |
| 17. Negativity/Pessimism | 0.02 | 0.22 | 0.13 | 0.06 | 0.08 | 0 | 0 | 0.4 | 0 | 0.03 | 0.04 | 0 | 0.06 | 0.04 | 0 | 0 | - |  |
| 18. Punitiveness | 0.12 | 0 | 0.03 | 0 | 0.23 | 0.1 | 0.02 | 0.02 | 0 | 0 | 0.04 | 0 | 0.15 | 0 | 0 | 0 | 0.1 | - |

Table S3. Partial correlation matrix from network analysis of early maladaptive schemas in patients with binge eating disorder.

|  | 1 | 2 | 3 | 4 | 5 | 6 | 7 | 8 | 9 | 10 | 11 | 12 | 13 | 14 | 15 | 16 | 17 | 18 |
| --- | --- | --- | --- | --- | --- | --- | --- | --- | --- | --- | --- | --- | --- | --- | --- | --- | --- | --- |
| 1. Emotional deprivation | - |  |  |  |  |  |  |  |  |  |  |  |  |  |  |  |  |  |
| 2. Abandonment | 0.06 | - |  |  |  |  |  |  |  |  |  |  |  |  |  |  |  |  |
| 3. Mistrust/Abuse | 0.17 | 0.16 | - |  |  |  |  |  |  |  |  |  |  |  |  |  |  |  |
| 4. Social isolation | 0.11 | 0 | 0.19 | - |  |  |  |  |  |  |  |  |  |  |  |  |  |  |
| 5. Defectiveness/Shame | 0.07 | 0.19 | 0 | 0.32 | - |  |  |  |  |  |  |  |  |  |  |  |  |  |
| 6. Failure | 0 | 0.04 | 0 | 0.12 | 0.07 | - |  |  |  |  |  |  |  |  |  |  |  |  |
| 7. Dependence/Incompetence | 0 | 0.05 | 0 | 0.05 | 0.1 | 0.33 | - |  |  |  |  |  |  |  |  |  |  |  |
| 8. Vulnerability to harm/Illness | 0 | 0.17 | 0 | 0.04 | 0 | 0 | 0.06 | - |  |  |  |  |  |  |  |  |  |  |
| 9. Enmeshment | 0 | 0 | 0 | 0 | -0.08 | 0.05 | 0.05 | 0.09 | - |  |  |  |  |  |  |  |  |  |
| 10. Subjugation | 0.12 | 0.21 | 0.04 | 0.17 | 0 | 0 | 0.23 | 0 | 0.2 | - |  |  |  |  |  |  |  |  |
| 11. Self-Sacrifice | 0.15 | 0.02 | 0.01 | 0 | -0.12 | 0 | -0.01 | 0.02 | 0.2 | 0.15 | - |  |  |  |  |  |  |  |
| 12. Emotional inhibition | 0.06 | -0.12 | 0.09 | 0.09 | 0.1 | 0 | 0 | -0.05 | 0 | 0.15 | -0.11 | - |  |  |  |  |  |  |
| 13. Unrelenting standards | 0.03 | 0.05 | 0.01 | 0 | 0 | 0 | -0.13 | 0 | 0 | -0.07 | 0.09 | 0.1 | - |  |  |  |  |  |
| 14. Entitlement/Grandiosity | 0.05 | 0 | 0.07 | 0.03 | 0.01 | -0.15 | 0 | 0 | 0 | 0 | 0 | 0.05 | 0.21 | - |  |  |  |  |
| 15. Insufficient self-control | 0 | 0 | 0.02 | 0 | 0.01 | 0.24 | 0.21 | 0 | 0.04 | 0 | -0.1 | 0.09 | 0 | 0.27 | - |  |  |  |
| 16. Approval seeking | 0 | 0.13 | 0.11 | 0.03 | 0.01 | 0 | 0 | 0.04 | 0 | 0 | 0 | 0 | 0.16 | 0.24 | 0.17 | - |  |  |
| 17. Negativity/Pessimism | 0 | 0.09 | 0.15 | 0 | 0 | 0.07 | 0 | 0.45 | 0.08 | 0.02 | 0 | 0.05 | 0.09 | 0 | 0.02 | 0 | - |  |
| 18. Punitiveness | 0 | 0.07 | 0 | 0 | 0.24 | 0.05 | 0 | 0 | 0 | 0 | 0.06 | 0 | 0.25 | 0 | 0 | 0.02 | 0.18 | - |

Table S4. Partial correlation matrix from network analysis of early maladaptive schemas in patients with obesity.

|  | 1 | 2 | 3 | 4 | 5 | 6 | 7 | 8 | 9 | 10 | 11 | 12 | 13 | 14 | 15 | 16 | 17 | 18 |
| --- | --- | --- | --- | --- | --- | --- | --- | --- | --- | --- | --- | --- | --- | --- | --- | --- | --- | --- |
| 1. Emotional deprivation | - |  |  |  |  |  |  |  |  |  |  |  |  |  |  |  |  |  |
| 2. Abandonment | 0 | - |  |  |  |  |  |  |  |  |  |  |  |  |  |  |  |  |
| 3. Mistrust/Abuse | 0.09 | 0.21 | - |  |  |  |  |  |  |  |  |  |  |  |  |  |  |  |
| 4. Social isolation | 0.17 | 0.18 | 0.32 | - |  |  |  |  |  |  |  |  |  |  |  |  |  |  |
| 5. Defectiveness/Shame | 0.15 | 0.24 | 0 | 0.18 | - |  |  |  |  |  |  |  |  |  |  |  |  |  |
| 6. Failure | 0 | 0 | 0 | 0.12 | 0.09 | - |  |  |  |  |  |  |  |  |  |  |  |  |
| 7. Dependence/Incompetence | 0 | 0 | 0 | 0 | 0.03 | 0.21 | - |  |  |  |  |  |  |  |  |  |  |  |
| 8. Vulnerability to harm/Illness | 0.04 | 0.05 | 0.03 | 0 | 0.03 | 0 | 0 | - |  |  |  |  |  |  |  |  |  |  |
| 9. Enmeshment | 0 | 0.07 | 0 | 0.04 | 0 | 0.17 | 0.22 | 0.1 | - |  |  |  |  |  |  |  |  |  |
| 10. Subjugation | 0 | 0.18 | 0.02 | 0.09 | 0.12 | 0.05 | 0.14 | 0.19 | 0.16 | - |  |  |  |  |  |  |  |  |
| 11. Self-Sacrifice | 0.09 | 0.07 | 0.11 | 0 | 0 | 0 | 0 | 0 | 0.05 | 0 | - |  |  |  |  |  |  |  |
| 12. Emotional inhibition | 0.07 | 0 | 0.12 | 0 | 0.01 | 0 | 0 | 0.07 | 0 | 0.07 | 0 | - |  |  |  |  |  |  |
| 13. Unrelenting standards | 0 | 0 | 0.07 | 0 | 0 | 0 | 0 | 0.04 | 0 | 0 | 0.19 | 0.06 | - |  |  |  |  |  |
| 14. Entitlement/Grandiosity | 0.11 | 0 | 0.11 | 0.07 | 0 | 0 | 0 | 0 | 0 | 0 | 0.11 | 0 | 0.23 | - |  |  |  |  |
| 15. Insufficient self-control | 0 | 0.01 | 0 | 0.01 | 0 | 0.28 | 0.09 | 0 | 0.05 | 0.09 | 0.01 | 0.09 | 0 | 0.16 | - |  |  |  |
| 16. Approval seeking | 0 | 0 | 0 | 0 | 0 | 0 | 0.08 | 0 | 0.12 | 0.01 | 0 | 0.06 | 0.11 | 0.23 | 0.08 | - |  |  |
| 17. Negativity/Pessimism | 0 | 0 | 0 | 0 | 0 | 0 | 0 | 0.34 | 0 | 0 | 0 | 0.16 | 0.03 | 0.02 | 0 | 0.12 | - |  |
| 18. Punitiveness | 0 | 0 | 0 | 0 | 0 | 0 | 0 | 0 | 0 | 0 | 0 | 0 | 0.14 | 0 | 0 | 0 | 0.54 | - |
